# Letter is in response to a published manuscript, “Quality of the Information provided by ChatGPT for patients in breast plastic surgery: Are we already in the future?”

**DOI:** 10.1016/j.jpra.2024.09.006

**Published:** 2024-09-19

**Authors:** Abdul Rahman, Irfan Alam

**Affiliations:** School of Computing Science Engineering and Artificial Engineering, VIT Bhopal University, Sehore 466114, India

Dear Editor,

We are writing this letter to express opinions on the recently published article, **“Quality of the Information Provided by ChatGPT for Patients in Breast Plastic Surgery: Are We Already in the future?”**.^1^ The integration of AI in healthcare, particularly in providing information for patients considering breast plastic surgery, is both a fascinating and crucial development.

The article^1^ brings to light several important points regarding the accuracy, accessibility, and potential of AI-driven tools like ChatGPT in delivering medical information. Here, We would like to share a few ideas that could further enhance this discussion and the future of AI in patient education.

Firstly, while the article rightly emphasizes the accuracy of information provided by ChatGPT, it would be beneficial to delve deeper into the methods used to validate this information. Ensuring that AI systems adhere to the latest medical guidelines and protocols is essential for maintaining trust and reliability.^2^ Regular updates and collaborations with medical professionals could be a way to keep the information current and accurate.

Secondly, the accessibility of ChatGPT for patients across different demographics is another area worth exploring. The user interface, language options, and cultural sensitivity of the information provided can significantly impact its usability. Making AI tools more inclusive and accessible to non-English speakers and those with varying levels of health literacy would broaden their reach and utility.

Moreover, the ethical implications of relying on AI for medical information cannot be overlooked. The potential for misinformation and the need for oversight by qualified healthcare providers are crucial considerations. Establishing clear guidelines on the role of AI in patient education and ensuring that patients are aware of the limitations and complementary nature of AI tools is paramount.

Lastly, the future of AI in healthcare, as suggested by the article, indeed looks promising. However, ongoing research and continuous improvement are necessary to address the evolving needs of patients and the healthcare landscape. Encouraging interdisciplinary collaborations between technologists, medical professionals, and patient advocacy groups will be key to realizing the full potential of AI in patient care.

In conclusion, the article provides a comprehensive overview of the current state of AI in breast plastic surgery patient education. By addressing the accuracy, accessibility, ethical considerations, and future directions, we can work towards an AI-enhanced healthcare system that benefits all patients.

Thank you for considering this letter on referred article, We look forward to further discussions and advancements in this field.

## Ethical approval

Not applicable.

## Availability of supporting data

There is no new data generated.

## Declaration of competing interest

Authors declare no conflict of interest.
